# Tertiary Lymphoid Structures are Linked to Enhanced Antitumor Immunity and Better Prognosis in Muscle‐Invasive Bladder Cancer

**DOI:** 10.1002/advs.202410998

**Published:** 2024-12-30

**Authors:** Jiaxing Lin, Shan Jiang, Baoqiang Chen, Yiqing Du, Caipeng Qin, Yuxuan Song, Yun Peng, Mengting Ding, Jilin Wu, Yihan Lin, Tao Xu

**Affiliations:** ^1^ Department of Urology Peking University People's Hospital Beijing 100044 China; ^2^ Center for Quantitative Biology and Peking‐Tsinghua Center for Life Sciences Academy for Advanced Interdisciplinary Studies, Peking University Beijing 100871 China; ^3^ The MOE Key Laboratory of Cell Proliferation and Differentiation, School of Life Sciences Peking University Beijing 100871 China; ^4^ Peking University Chengdu Academy for Advanced Interdisciplinary Biotechnologies Chengdu Sichuan 610213 China

**Keywords:** B cells, CXCL13, muscle‐invasive bladder cancer, tertiary lymphoid structures

## Abstract

The prognosis for muscle‐invasive bladder cancer (MIBC) remains poor, and reliable prognostic markers have yet to be identified. Tertiary lymphoid structures (TLS) have been associated with favorable outcomes in certain cancers. However, the relationship between TLS and MIBC remains unclear. A multi‐omics approach is utilized, leveraging single‐cell RNA sequencing, spatial transcriptomics, bulk RNA sequencing, and immunohistochemistry, to investigate the roles of B cells and TLS in MIBC. These findings indicate that elevated levels of B cells and TLS correlate with improved prognoses in patients with MIBC, aligning with the robust antitumor immune responses observed in the TLS region. From a mechanistic perspective, CXCL13 serves as a critical cytokine for TLS formation in MIBC, primarily secreted by clonally expanded CXCL13+ T cells. This cytokine interacts with the CXCR5 receptor on NR4A2+ B cells, promoting TLS development. Plasma cells arising within the TLS microenvironment predominantly produce the IGHG antibody, potentially enhancing the phagocytic capabilities of C1QC+ macrophages. From an application standpoint, a TLS‐specific gene signature is developed that effectively predicts outcomes in MIBC and other cancers. This study highlights the prognostic potential of TLS in MIBC and reveals immune mechanisms, offering insights for personalized treatment strategies.

## Graphical abstract

This study employed multi‐omics approaches to investigate the role of B cells and TLS in muscle‐invasive bladder cancer (MIBC) and their potential clinical applications. The results indicate that both B cells and TLS are associated with improved prognosis and enhanced antitumor immunity in MIBC patients. Furthermore, the TLS biomarker developed can robustly predict the prognosis of MIBC.

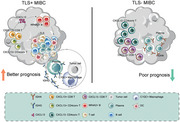

## 1 Introduction

Urothelial carcinoma (UC) is a prevalent and lethal malignancy of the urinary system. Muscle‐invasive bladder cancer (MIBC), a clinical subtype of UC, is associated with a poor prognosis and limited treatment options.^[^ ^1^ ^]^ The primary treatments for MIBC currently include cisplatin‐based neoadjuvant chemotherapy and radical cystectomy, yet the overall 5‐year survival rate remains only 60–70%.^[^ ^2^ ^]^ Recently, immune checkpoint inhibitors (ICB), such as anti‐PD‐1/PD‐L1 antibodies, have demonstrated significant efficacy in treating MIBC, particularly in patients with advanced disease. However, only a subset of patients exhibited a durable response to immunotherapy, underscoring the critical need for new biomarkers to more accurately predict therapeutic response.

The presence of tertiary lymphoid structures (TLS) has been associated with improved clinical outcomes and enhanced responses to immunotherapy in several cancers.^[^ ^3^, ^4^, ^5^ ^]^ TLS are organized aggregates of immune cells that form in non‐lymphoid tissues,^[^ ^6^ ^]^ with B cells being the predominant cell type. Some studies have examined the prognostic significance of TLS in UC cohorts. Research suggests that the clustering of highly expressed TLS‐related genes is associated with a better prognosis.^[^ ^7^ ^]^ In one cohort of UC patients treated with combined CTLA‐4 and PD‐1 inhibitors, responders exhibited higher baseline TLS density.^[^ ^8^ ^]^ However, in another cohort receiving similar treatment, baseline TLS did not significantly affect prognosis, likely due to corticosteroid use, which may have inhibited TLS formation.^[^ ^9^ ^]^ In non‐immunotherapy cohorts, TLS have been shown to predict both progression‐free survival and overall survival in UC patients.^[^ ^10^, ^11^ ^]^ Although several studies have investigated the prognostic value of TLS in MIBC, sample sizes, and cohort numbers have been relatively small compared to those in other cancers,^[^ ^4^ ^]^ highlighting the need for further validation and investigation.

Beyond its prognostic significance, the complex mechanistic role of TLS in tumors is a critical area of tumor immunology research. For instance, studies have shown that the presence of TLS and tissue‐resident T cells within tumors can promote the efficacy of anti‐PD‐1 therapy in gastric cancer.^[^ ^12^ ^]^ In esophageal squamous cell carcinoma, Tfh cells within TLS express SEMA4D, which stimulates dendritic cells and enhances their maturation and antigen‐presenting capabilities.^[^ ^13^ ^]^ In hepatocellular carcinoma, reduced intra‐tumoral TLS abundance has been linked to mTOR signaling activation and dysregulated cell cycle progression, both associated with poor prognosis.^[^ ^14^ ^]^ However, the potential anti‐tumor mechanisms of TLS in MIBC remain unclear.

**Figure 1:**
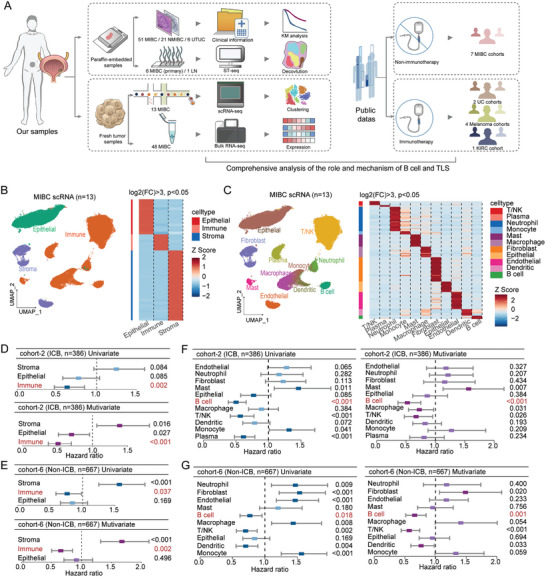
Cox regression analysis of cell subpopulations. A) The workflow diagram illustrates the data collection process, with in‐house data on the left and public data on the right. B) The UMAP plot shows the clustering of cells from 13 MIBC samples, grouped into three major categories. The accompanying heatmap highlights the expression of characteristic genes within these cell types. FC, fold change. C) Subpopulations. D,E) Forest plots of univariate and multivariate Cox regression analyses for major subpopulations. F,G) Forest plots for smaller subpopulations analyzed using univariate and multivariate Cox regression.

This study aims to systematically investigate the role of B cells and TLS in the MIBC tumor microenvironment through a multi‐omics approach by integrating single‐cell RNA sequencing (scRNA‐seq), spatial transcriptomics (ST), and bulk RNA‐seq (**Figure** **1**A). We investigated the impact of B cell and TLS abundance on patient prognosis, elucidating the cellular composition and potential functions of TLS within MIBC tumors. Meanwhile, we explored the interactions of TLS within the MIBC tumor microenvironment. Finally, we developed a specific set of markers for TLS that can accurately predict the prognosis of MIBC. Our findings underscore the potential of B cells and TLS as biomarkers and enhance our understanding of the potential immunoregulatory mechanisms of TLS in MIBC.

## 2 Results

### 2.1 Cell Abundance Serves as a Predictive Marker for Prognosis in UC Patients Undergoing ICB Therapy as well as in MIBC Patients not Receiving ICB Treatment

To identify potential cell types relevant to prognosis, we conducted scRNA‐seq on fresh tumor samples from 13 MIBC patients treated at our hospital, including six cases from previously published datasets.^[^ ^15^ ^]^ Through dimensionality reduction and clustering, we first categorized the cells into three main groups: epithelial cells, immune cells, and stromal cells (Figure 1B). Further analysis identified distinct subpopulations, including T/NK cells, plasma cells, neutrophils, monocytes, mast cells, macrophages, dendritic cells, B cells, fibroblasts, endothelial cells, and epithelial cells (Figure 1C). Differential expression analysis revealed specific marker genes for these subpopulations (log2 FC > 3, *p* < 0.05).

Subsequently, we categorized the public transcriptomic cohorts based on whether ICB therapy had been administered, combining cohort‐2 UC (ICB, *n* = 386) and cohort‐6 MIBC (Non‐ICB, *n* = 667). Using the MCP‐counter tool and identified markers from the above analysis, we next quantitatively assessed the abundance of various cell types in cohort‐2 and cohort‐6. Univariate and multivariate Cox regression analyses of major subpopulations (Figure 1D,E) demonstrated that the presence of immune cells significantly correlated with survival in cohort‐2 and cohort‐6, while stromal cells showed an opposite association. Similar analysis applied to smaller subpopulations (Figure 1F,G) revealed that cohort‐2, B cells and T/NK cells may contribute to improved prognoses, whereas mast cells were associated with increased risk, with B cells displaying the lowest hazard ratio (HR = 0.5), thereby halving the risk. Additionally, in cohort‐6, B cells, T/NK cells, and dendritic cells demonstrated a favorable prognostic effect, whereas fibroblasts were associated with poorer outcomes. These findings suggest that B cells may play a critical prognostic role in the tumor microenvironment of MIBC, potentially independent of other cell types. Kaplan–Meier analysis further confirmed that patients with higher B cell levels had improved prognoses (Figure S1A,B, Supporting Information). Moreover, the expression levels of the key B cell markers, CD19 and CD20, can also predict patient outcomes (Figure S1C, Supporting Information). Meanwhile, B cells were more abundant in female patients and in patients with high‐grade disease (Figure S1D, Supporting Information). To explore the potential contribution from other immune cell types, we performed co‐expression analysis and found that B cells were most closely correlated with T/NK cell abundance (Figure S1E, Supporting Information). Consistently, patients with elevated B cell levels showed upregulation of immune‐related pathways, such as pathway for “T cell activation” (Figure S1F, Supporting Information).

### 2.2 The Abundance of TLS can Predict the Prognosis of UC Patients at our Hospital

**Figure 2:**
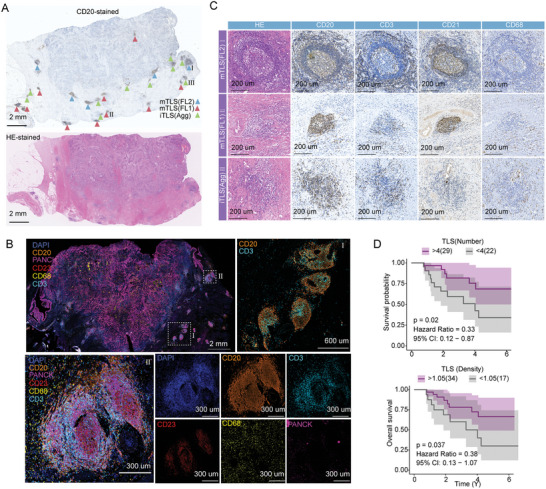
TLS staining and clinical analysis of hospital specimens. A) Panoramic view of CD20 and HE staining, illustrating the distribution of TLS in MIBC samples and highlighting TLS of varying maturity, including iTLS, FL1, and FL2. B)Multicolor  IF staining of MIBC samples, including DAPI, CD20, PANCK, CD23, CD68, and CD3. C) Staining results for TLS at different maturity stages using HE, CD20, CD3, CD21, and CD68. D) Kaplan–Meier survival analysis showing the impact of TLS number and density on UC patient prognosis.

Because the above analyses based on public cohorts suggested the prognostic potential of B‐cells, we sought to substantiate this conclusion by analyzing samples from patients treated at our hospital. We collected paraffin‐embedded specimens from 51 MIBC, 21 non‐muscle‐invasive Bladder Cancer (NMIBC), and 6 upper tract urothelial carcinoma (UTUC) patients. These samples underwent hematoxylin and eosin (HE) staining and immunohistochemistry (IHC) for CD20, CD3, CD21, and CD68, key immune cell markers. We observed that B cells aggregate within certain areas, forming TLS (**Figure** **2**A). We validated the presence of TLS using multiplex immunofluorescence (IF) (Figure 2B), revealing that CD20 was highly concentrated, CD3 was dispersed within TLS, and CD68 was present both within and around TLS. TLS was abundant in urothelial carcinoma, with higher levels in MIBC and UTUC (Figure S2A, Supporting Information). In MIBC, TLS of varying maturity were identified (Figure 2C), including iTLS (immature TLS) and mTLS (mature TLS), with mTLS further classified into FL1 (Primary follicle) and FL2 (Secondary follicle). iTLS comprised 55.9% of the TLS in MIBC, FL1 37.6%, and FL2 6.5% (Figure S2B, Supporting Information). Analysis of clinical characteristics revealed that TLS density was significantly higher in high‐grade UC (Figure S2C, Supporting Information). Based on HE and IHC results, sample were categorized into tumor regions and stromal regions (Figure S2D, Supporting Information), with TLS more likely to accumulate in the stromal region (Figure S2E, Supporting Information), accounting for 55.2%. Moreover, TLS in the stromal region tended to be more mature (Figure S2F, Supporting Information). Finally, we assessed the prognostic value of TLS in UC patients, finding that the number or density of TLS could predict patient prognosis (Figure 2D). Survival analysis based on mTLS density in the stromal region suggested that patients with higher stromal TLS density tended to have better prognoses, although this was not statistically significant (Figure S2G, Supporting Information).

### 2.3 Spatial Transcriptomics Reveals Cellular Heterogeneity Between TLS‐Area and no TLS‐Area

**Figure 3:**
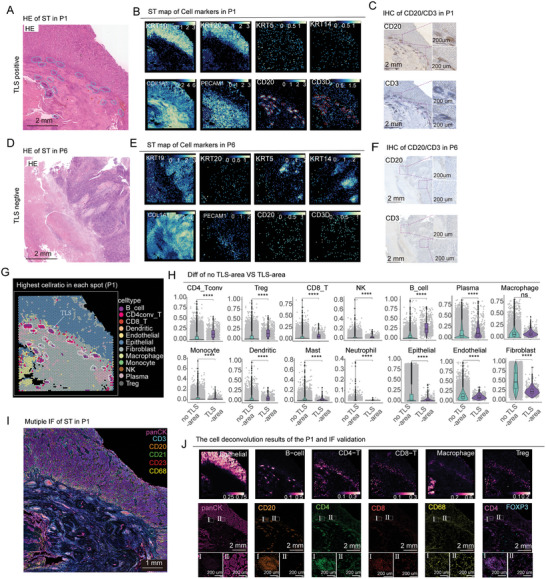
Analysis of cellular heterogeneity between TLS and non‐TLS areas in bladder cancer. A) HE staining highlights the TLS‐rich region in the P1 sample. B) Spatial expression maps of various cell marker genes, including KRT19, KRT20, KRT5, KRT14, COL1A1, PECAM1, CD20, and CD3, in the P1 sample. C) IHC staining for CD20 and CD3 in the P1 sample. D) HE staining of the P6 sample. E) Spatial expression of marker genes in the P6 sample. F) IHC staining for CD20 and CD3 in the P6 sample. G) Distribution of different cell types in the P1 ST. H) Comparison of cell content between TLS‐area and no TLS‐area. I) Multicolor immunofluorescence staining of the P1 sample. J) The upper panel shows the cell deconvolution results for the P1 sample, while the lower panel shows the corresponding staining for cell markers. “I” represents the TLS‐area, and “II” represents the no TLS‐area. “****” indicates *p* < 0.0001.

To investigate the cellular composition and function of TLS in MIBC, we conducted spatial transcriptomics sequencing on seven paraffin‐embedded MIBC samples. Among these, P1, P3 to P5 were TLS‐positive (TLS‐pos), P6 to P7 were TLS‐negative (TLS‐neg), and P2 was a lymph node metastasis sample. HE staining of the P1 sample revealed abundant TLS presence (**Figure** **3**A), and spatial gene expression analysis identified this sample as belonging to the luminal subtype (Figure 3B), where KRT19 was used as a marker for bladder cancer, KRT20 for the luminal subtype, KRT5 and KRT14 for the basal subtype,^[^ ^16^ ^]^ COL1A1 served as a marker for fibroblasts, PECAM1 for endothelial cells, CD20 for B cells, and CD3 for T cells. Moreover, high expression levels of CD20 and CD3 genes were observed specifically in the TLS region as expected. Subsequent IHC staining of adjacent ST sections for CD20 and CD3 further validated the capacity of spatial transcriptomics to accurately characterize TLS (Figure 3C). Conversely, the P6 sample, identified as TLS‐neg (Figure 3D), showed gene expression consistent with the basal subtype and lacked clustered CD20 and CD3 expression (Figure 3E). IHC staining of the P6 sample also confirmed the absence of TLS (Figure 3F). Additionally, IHC validation was performed on four other samples (Figure S3A–C, Supporting Information).

Using SpaCET combined with single‐cell data from 13 cases, we conducted deconvolution analysis on the ST samples (Figure 3G). The deconvolution results showed significant enrichment of CD4+ conventional T (CD4conv T), regulatory T cells (Tregs), CD8+ T cells, NK cells, B cells, plasma cells, and dendritic cells within TLS areas (Figure 3H), while monocytes, mast cells, neutrophils, fibroblasts, endothelial cells, and epithelial cells were more prevalent in non‐TLS areas. Furthermore, multicolor IF on the P1 sample (Figure 3I,J) revealed staining patterns closely aligned with the deconvolution results, reinforcing the validity of our findings. The TLS region indeed exhibited higher levels of CD20+ B cells, CD4+ T cells, CD8+ T cells, and Treg cells, along with fewer epithelial cells.

### 2.4 TLS Exhibits Stronger Anti‐Tumor Immune Responses

**Figure 4:**
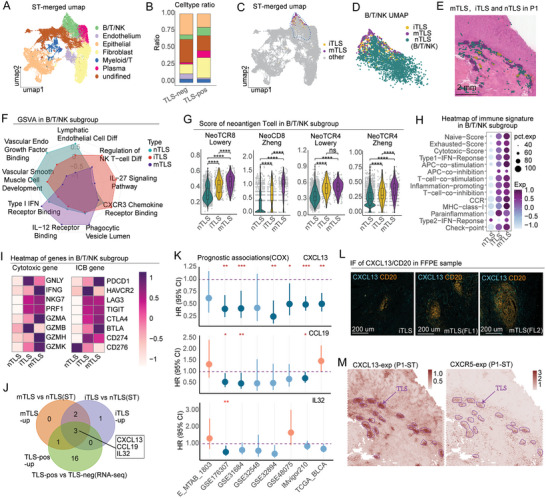
Functional analysis of TLS. A) UMAP plot integrating six ST samples. B) Proportion analysis of subpopulations, categorized by TLS‐pos and TLS‐neg groups. C) Mapping the distribution of TLS on the UMAP. D) Identification of B/T/NK subpopulations. E) Visualization of mTLS, iTLS, and nTLS in the P1 sample. F) Radar chart presenting the GSVA results. G) Comparison of tumor‐reactive T cell scores. H) Heatmap depicting immune‐related pathway score. I) Heatmap showing the expression of cytotoxic and ICB‐related genes. J) A Venn diagram illustrating the differentially expressed genes. K) Results of univariate Cox analysis for CXCL13, IL32, and CCL19 across eight independent cohorts. L) Immunofluorescence staining of CXCL13 and CD20 in FFPE samples. M) Spatial expression patterns of CXCL13 and CXCR5 in ST data. *p < 0.05, **p < 0.01, ***p < 0.001, ****p < 0.0001.

To further analyze the function of TLS, we integrated six ST samples of primary MIBC. We used the Harmony method to correct for batch effects (Figure S4A,B, Supporting Information). The cell subpopulations were annotated and classified into seven distinct groups (**Figure** **4**A; Figure S4D, Supporting Information). The Plasma subgroup predominantly expressed high levels of genes characteristic of plasma cells (Figure S4C, Supporting Information), while the Myeloid/T subgroup displayed gene expression profiles typical of myeloid cells and T cells. The B/T/NK subgroup primarily showed co‐expression of characteristic genes for T cells, NK cells, and B cells. Analysis of cell proportions revealed that in the TLS‐pos group, the proportions of B/T/NK cells, and plasma cells were significantly increased, whereas endothelial cells were more prevalent in the TLS‐neg group (Figure 4B). Combining CD21 gene expression with IHC staining, we categorized TLS in the ST samples into iTLS and mTLS (Figure S4E,F, Supporting Information). UMAP analysis revealed that TLS predominantly clustered in the B/T/NK cell region (Figure 4C), with notable distribution differences. We defined the B/T/NK cell region outside TLS as nTLS (non‐TLS, Figure 4D) and mapped these regions back onto the ST map, finding that nTLS primarily surrounded the TLS (Figure 4E; Figure S4G, Supporting Information). The GSVA analysis of mTLS, iTLS, and nTLS (Figure 4F) revealed that mTLS was significantly enriched for “Type 1 IFN Receptor Binding,” suggesting that mTLS may play a role in antitumor immunity. In nTLS, enrichment of lymphangiogenesis and angiogenesis pathways suggests that immune cells are being recruited within this immune cluster, facilitating its development toward TLS formation. To enable additional characterization, we curated markers for tumor‐reactive T cells from two studies.^[^ ^17^, ^18^ ^]^ Using these markers, we scored the tumor‐reactive T cells within the TLS (Figure 4G) and found that mTLS had the highest tumor‐reactive scores for CD4 and CD8 T cells, while nTLS had the lowest. Further analysis of immune signaling pathways (Figure 4H) revealed that both mTLS and iTLS exhibited significant upregulation of cytotoxic scores, exhaust score, chemokine receptor activity, and inflammation‐promoting pathways. In mTLS, there was a significant upregulation of IFNG, GZMA, GZMH, GZMK, PDCD1, and BTLA, whereas in iTLS, expression levels of GNLY, GZMB, and HAVCR2 were elevated. In contrast, nTLS exhibited increased expression of CD276 genes (Figure 4I). Immunofluorescence for CD4, CD8, and PDCD1 on the P1 sample confirmed the enrichment of PDCD1+ CD4 T cells in the mTLS and iTLS region (Figure S4H, Supporting Information).

We subsequently explored the underlying mechanisms of TLS formation. Data for 189 cytokines were retrieved from the UniProt database (Table S1, Supporting Information), and we conducted differential analyses between mTLS and nTLS (ST), iTLS and nTLS (ST), as well as between TLS‐pos and TLS‐neg samples (Bulk RNA‐seq). By intersecting the sets of differentially expressed genes (log2 FC > 0.3), we identified consistent upregulation of CXCL13, CCL19, and IL32 within the mTLS, iTLS, and TLS‐pos groups (Figure 4J). To further investigate the therapeutic potential of CXCL13, CCL19, and IL32 for urothelial carcinoma, we performed Cox survival analyses in eight distinct cohorts. Our results showed that CXCL13 had a hazard ratio (HR) below 1 across all cohorts, with *p*‐values less than 0.05 in six of these cohorts (Figure 4K). Notably, IF staining of CXCL13 verified its expression in TLS (Figure 4L). Moreover, significant expression of CXCL13 and its receptor CXCR5 was observed in TLS within the ST samples (Figure 4M). CXCL13 is a key chemokine for B cells and plays a crucial role in TLS formation.^[^ ^19^ ^]^ Previous studies have also highlighted the prognostic value of CXCL13 in bladder cancer^[^ ^20^ ^]^; however, these studies included only two cohorts. Our study, utilizing eight cohorts, demonstrates the potential clinical translational capability of CXCL13.

### 2.5 CXCL13+ T Cells are Upregulated in TLS‐Pos Samples, Correlating with a Stronger Anti‐Tumor Response

To further investigate the relationship between TLS and the tumor immune microenvironment, we categorized our hospital's scRNA‐seq samples into TLS‐pos and TLS‐neg groups based on the presence of TLS in the corresponding paraffin‐embedded samples (Figure S5A, Supporting Information). Four samples lacked TLS, while the remaining samples contained TLS, with at least one mTLS present (Figure S5B, Supporting Information). Dimensionality reduction and clustering revealed distinct differences in the proportions of cell subpopulations between the two groups (Figure S5C, Supporting Information). The TLS‐neg group was enriched with mast cells, and neutrophils, whereas the TLS‐pos group exhibited higher proportions of plasma cells, and B cells (Figure S5D, Supporting Information).

**Figure 5:**
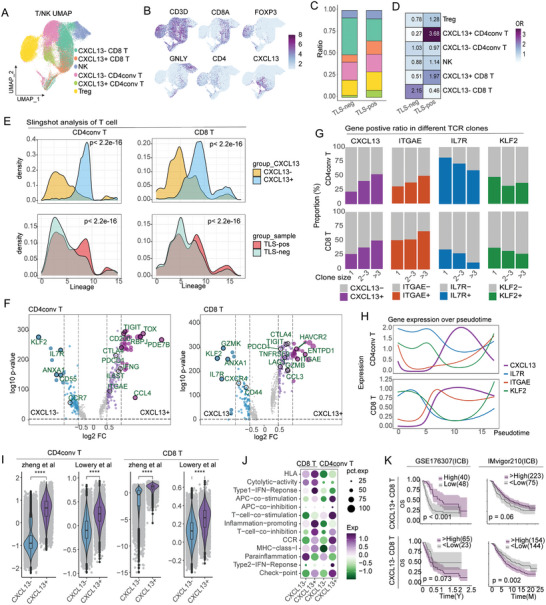
Analysis of T/NK cell subpopulations. A) UMAP plot depicting T/NK cell subpopulations. B) Expression levels of CD3D, CD8A, FOXP3, GNLY, CD4, and CXCL13 in T/NK cells. C) Comparison of the proportion of CXCL13+ cells between TLS‐pos and TLS‐neg samples. D) Analysis of tissue‐specific biases in cell subpopulations. E) Slingshot trajectory analysis of T cell development, grouped by CXCL13 expression and TLS status. F) Differential gene expression analysis between CXCL13+ and CXCL13‐ cells. G) Correlation between TCR clonality and the proportion of CXCL13, ITGAE, IL7R, and KLF2 positive cells. H) Gene expression trends for CXCL13, IL7R, KLF2, and ITGAE during T cell development. I) Tumor reactivity score comparison between CXCL13+ and CXCL13‐ T cells. J) Heatmap illustrating immune pathway scores in CXCL13+ and CXCL13‐ T cells. K) Kaplan–Meier survival analysis based on CXCL13 expression levels in CD8 T cells. ****p < 0.0001.

Since UMAP analysis revealed that CXCL13 was primarily expressed in the T/NK subpopulation (Figure S5E, Supporting Information), we focused our analysis on T/NK cells. We subsequently performed deconvolution analysis of T/NK cells in the ST samples and found a higher abundance of these cells in the TLS‐pos group (Figure S6A, Supporting Information). This finding was further validated using adjacent tissue sections. Drawing from previous studies,^[^ ^21^, ^22^ ^]^ the T/NK subpopulation was further subdivided into six groups (**Figure** **5**A,B). Both CXCL13+ CD4conv T cells and CXCL13+ CD8 T cells were significantly upregulated in the TLS‐pos group (Figure 5C,D). To analyze T cell evolution, slingshot trajectory analysis was performed (Figure S6B, Supporting Information), revealing that CXCL13+ T cells were more likely to appear in the later stages of cell development. In TLS‐pos samples, T cells tended to differentiate into CXCL13+ T cells (Figure 5E). To further explore the transcriptomic differences between CXCL13⁺ and CXCL13⁻ cells, differential expression analysis was performed on CD4conv T and CD8 T cells. The results showed that IL7R, KLF2, and ANXA1 were upregulated in CXCL13‐ CD4conv T cells, while TIGIT, TOX, RBPJ, and CCL4 were upregulated in CXCL13+ CD4conv T cells. Similarly, IL7R, KLF2, and ANXA1 were upregulated in CXCL13‐ CD8 T cells, whereas ITGAE, ENTPD1, and HAVCR2 were upregulated in CXCL13+ CD8 T cells. IL7R and KLF2 contribute to maintaining T cell quiescence,^[^ ^23^, ^24^ ^]^ while ENTPD1, ITGAE, and PDCD1 are markers of tumor‐reactive T cells.^[^ ^18^, ^25^, ^26^ ^]^ Gene trajectory analysis indicated that CXCL13 expression was initially low but significantly increased during cell development, whereas IL7R and KLF2 showed opposite expression patterns (Figure 5H). ITGAE was co‐expressed with CXCL13 in CD8 T cells, a pattern also observed in our bulk data (R = 0.64; Figure S6C, Supporting Information) and in lung cancer studies.^[^ ^27^ ^]^ Research showed that ITGAE+ CD8 T cells were upregulated in TLS‐pos gastric cancer.^[^ ^12^ ^]^ In our samples, ST and IF staining revealed that ITGAE+ CD8 T cells were abundant in TLS‐pos MIBC (Figure S6D,E, Supporting Information). To further investigate the relationship between these markers and the antitumor response of T cells, we used TRUST4 to predict TCR clones and categorized them into three levels: 1, 2–3, and greater than 3. As clonal expansion increased, the positivity rates of CXCL13 and ITGAE significantly increased, while those of IL7R and KLF2 decreased (Figure 5G). Clonal expansion, representing the generation of tumor‐reactive T cells, suggests that CXCL13 and ITGAE are potential markers for tumor‐reactive T cells in MIBC. To further validate the hypothesis that CXCL13⁺ T cells are tumor‐reactive, we scored T cells for tumor‐reactive markers and found that CXCL13⁺ T cells exhibited significantly higher reactivity compared to CXCL13⁻ T cells (Figure 5I). Immune‐related pathway analysis revealed that check‐point, CCR, and APC‐co‐stimulation pathways were upregulated in CXCL13+ T cells, while para‐inflammation was downregulated (Figure 5J). Finally, using differential genes from four cell types for MCP scoring and combining survival data for KM analysis, we found that samples with high CXCL13+ CD8 T cells were associated with better patient prognosis, while CXCL13‐ CD8 T cells had the opposite effect (Figure 5K; Figure S6F, Supporting Information). However, no obvious differences were observed between CXCL13+ CD4conv T and CXCL13‐ CD4conv T cells (Figure S6G, Supporting Information). Our results suggest that CXCL13+ CD8 T cells are upregulated and undergo clonal expansion in TLS‐pos samples, potentially contributing to improved patient outcomes.

### 2.6 CXCL13 is Likely to Target the CXCR5 on NR4A2 B Cells, Thereby Catalyzing the Formation of TLS in MIBC

The interaction between CXCL13 and CXCR5 constitutes a critical pathway for TLS development.^[^ ^19^ ^]^ We conducted an analysis of CXCR5 expression, identifying its primary expression on T/NK and B cells (Figure S7A, Supporting Information). Subsequently, we classified B cells into distinct subgroups: DUSP4 Atm B (Atypical memory B), ITGB1 SwBm (Switched memory B), NR4A2 B, PTPRJ B, and TCL1A naive B (Figure S7B,C, Supporting Information). Comparative analysis of TLS‐neg and TLS‐pos samples revealed a higher prevalence of NR4A2 B cells in the TLS‐pos group (Figure S7D, Supporting Information). Further, CXCR5 exhibited increased expression specifically within NR4A2 B cells (Figure S7E, Supporting Information), an upregulation of CXCR5 was observed in TLS‐pos samples (Figure S7F, Supporting Information). Spatial distribution patterns showed that NR4A2 B cells, along with CXCL13+ CD4conv T cells and CXCL13+ CD8 T cells, were predominantly found in TLS regions (Figure S7G, Supporting Information). Notably, in TLS‐pos samples, co‐localization occurred between NR4A2 B cells and either CXCL13+ CD4conv T cells or CXCL13+ CD8 T cells (Figure S7H, Supporting Information). These findings imply that CXCL13 may bind to CXCR5 on NR4A2 B cells in MIBC, promoting the formation of TLS.

### 2.7 Plasma Cells in TLS‐Pos Samples Secrete More IGHG, Correlating with Improved Prognosis

**Figure 6:**
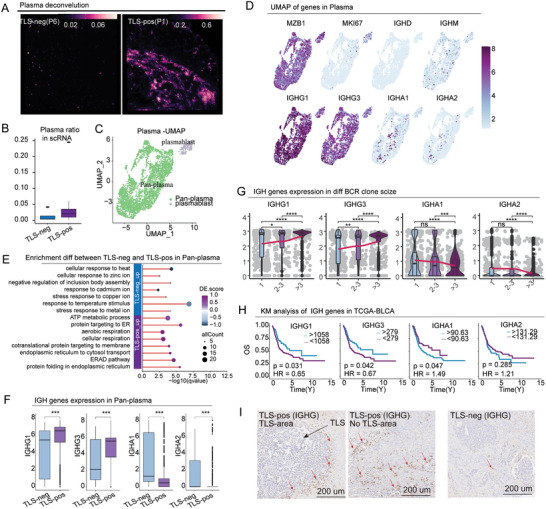
Plasma cell analysis. A) Convolution results of plasma cells in ST samples. B) Proportional analysis of plasma cells. C) UMAP plot depicting plasma cell subgroups. D) Expression levels of key plasma cell marker genes (MZB1, MKI67, IGHD, IGHM, IGHG1, IGHG3, IGHA1, IGHA2). E) Pathway enrichment analysis of pan‐plasma cells. F) Differential gene expression of IGHG and IGHA. G) Expression patterns of IGH genes across different BCR clone groups. H) Survival analysis based on IGH gene expression in the TCGA‐BLCA cohort. (I) IHC staining images of IGHG. ***p < 0.001, ****p < 0.0001.

Some B cells in mature TLS differentiate into plasma cells, producing antibodies that induce antitumor immunity.^[^ ^28^ ^]^ Thus, we aimed to investigate the differences in plasma cells between TLS‐pos and TLS‐neg MIBC samples. Both the deconvolution results of plasma cells (**Figure** **6**A) and the proportion of plasma cells (Figure 6B) demonstrate that plasma cells are more abundant in TLS‐pos samples. Our analysis identified two distinct plasma cell subgroups: plasmablasts and pan‐plasma (Figure 6C). In MIBC, plasma cells primarily express IGHG, with IGHA being expressed to a lesser extent (Figure 6D). Differential expression (DE) score analysis of the pan‐plasma subgroup revealed that antibody production‐related signaling pathways are enriched in TLS‐pos samples, while stress‐related pathways are more prevalent in TLS‐neg samples (Figure 6E). Therefore, we aimed to examine whether there are differences in antibody expression between the two groups. The results revealed that IGHG1 and IGHG3 were significantly upregulated in TLS‐pos samples, while IGHA1 and IGHA2 were upregulated in TLS‐neg samples (Figure 6F). The number of clones is indicative of tumor reactivity, prompting us to examine the differences in IGH clone numbers between the two groups. Using TRUST4 to predict BCR clones, we identified an intriguing pattern: samples with higher clonal expansion (>3) exhibited upregulation of IGHG1 and IGHG3, while IGHA1 and IGHA2 were downregulated (Figure 6G). This finding suggests that IGHG antibodies are the primary anti‐tumor antibodies produced by plasma cells within the tumor microenvironment. Survival analysis of IGH genes in the TCGA‐BLCA cohort further demonstrated that high expression of IGHG1 and IGHG3 is associated with better prognosis, while high expression of IGHA1 and IGHA2 correlates with poorer outcomes (Figure 6H). To conclude, we conducted IHC staining for IGHG and observed a significant abundance of IGHG expression in TLS‐pos samples, while its presence was notably diminished in TLS‐neg samples (Figure 6I).

Given that antibodies in the tumor microenvironment can mediate antibody‐dependent cellular phagocytosis (ADCP) by macrophages, we further analyzed macrophage subpopulations.^[^ ^29^ ^]^ We categorized myeloid cells into 11 subgroups, including 4 macrophage subpopulations (Figure S8A, Supporting Information). All macrophage subpopulations were found to be more enriched in TLS‐pos samples (Figure S8B, Supporting Information). CD68 is a marker for tumor‐infiltrating macrophages. IHC analysis confirmed that TLS‐pos MIBC tumors contain a higher density of macrophages (Figure S8C,D, Supporting Information). Phagocytic function scoring revealed that the Macro_C1Q1 subgroup exhibited significantly increased phagocytic activity in TLS‐pos samples (Figure S8E, Supporting Information). Macrophages are functionally classified into the tumor‐suppressive M1 phenotype and the tumor‐promoting M2 phenotype, with the potential for phenotypic switching between the two.^[^ ^30^ ^]^ Polarization analysis of Macro_C1Q1 revealed that these cells are more likely to polarize toward the M1 subtype in TLS‐pos samples, whereas they tend to polarize toward the M2 subtype in TLS‐neg samples (Figure S8F, Supporting Information). Our findings suggest that in TLS‐pos MIBC, plasma cells secrete higher levels of IGHG, which may enhance the phagocytic activity of C1QC+ macrophages, thereby amplifying the antitumor immune response.

### 2.8 TLS Markers can Predict Prognosis in MIBC and Other Tumor Immunotherapy Cohorts

**Figure 7:**
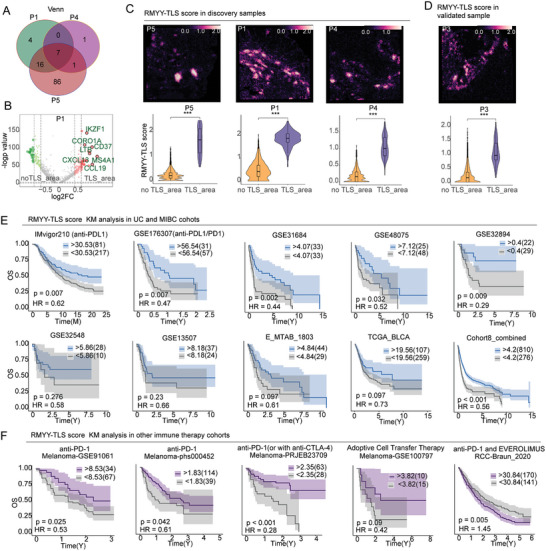
Construction of the TLS marker RMYY‐TLS and its prognostic value evaluation. A) Venn diagram illustrating the overlap of differential genes across various ST samples. B) Volcano plot comparing differential gene expression between no TLS‐area and TLS‐area. C) The upper panel shows the distribution of RMYY‐TLS scores in the discovery samples, while the lower panel compares RMYY‐TLS scores between the two areas. D) Validation sample results. E) Survival analysis based on RMYY‐TLS scores in UC and MIBC patients. F) Survival analysis of RMYY‐TLS scores in additional immunotherapy cohorts, including those with melanoma and renal cell carcinoma. ***p < 0.001.

Identifying representative markers for TLS is a central focus in research^[^ ^6^ ^]^; however, no TLS‐specific markers have been identified for MIBC to date. We used samples P1, P4, and P5 as the discovery samples, and sample P3 as the validation sample, to identify TLS signature markers through differential analysis of TLS‐area and no TLS‐area. This differential analysis revealed seven common differential genes (*p* < 0.05, logFC > 0.7) (**Figure** **7**A), including CXCL13, CCL19, MS4A1, LTB, CD37, CORO1A, and IKZF1 (Figure 7B; Figure S9A, Supporting Information), collectively termed RMYY‐TLS. Utilizing MCP and RMYY‐TLS for evaluation in ST, we found that RMYY‐TLS effectively differentiated between TLS‐area and no TLS‐area in both discovery and validation samples (*p* < 0.001, Figure 7C,D). To further validate the presence of TLS, we analyzed datasets from renal cell carcinoma, hepatocellular carcinoma, and nasopharyngeal carcinoma to assess the capacity of RMYY‐TLS to distinguish TLS‐area (Figure S9B, Supporting Information). In the ST samples, areas with high RMYY‐TLS scores consistently overlapped with TLS regions identified through corresponding HE staining (Figure S9C,D, Supporting Information).

Subsequently, we applied MCP and RMYY‐TLS scoring to two ICB UC cohorts and seven no ICB MIBC cohorts, followed by survival analysis using clinical data (Figure 7E). The results showed that RMYY‐TLS could predict tumor prognosis in both ICB cohorts (IMvigor210 and GSE176307) (*p* < 0.05) and in non‐ICB cohorts (GSE31684, GSE48075, and GSE32894). Although not statistically significant, the remaining four cohorts (GSE32548, GSE13507, E_MTAB_1083, TCGA_BLCA) displayed a trend where higher RMYY‐TLS levels correlated with better prognosis. When these cohorts were combined, patients with high RMYY‐TLS levels had significantly improved prognoses (*p* < 0.001). BCG therapy is a widely used immunotherapy for NMIBC cancer. Accordingly, we applied RMYY‐TLS to assess the prognostic outcomes in a BCG‐treated cohort. Although the trend did not reach statistical significance, patients with high RMYY‐TLS scores still exhibited a trend toward improved prognosis (Figure S9E,F, Supporting Information). Our findings suggest that RMYY‐TLS possesses superior predictive ability in patients receiving immunotherapy, prompting further examination across five additional immunotherapy cohorts. In melanoma, a representative hot tumor, patients with high RMYY‐TLS levels derived significant benefit from immunotherapy in four melanoma cohorts (Figure 7F). Conversely, in renal cell carcinoma, a representative cold tumor, higher RMYY‐TLS levels were associated with poorer prognosis (Figure 7F). Our results indicate that TLS markers have potential clinical application value.

## 3 Discussion

**Figure 8:**
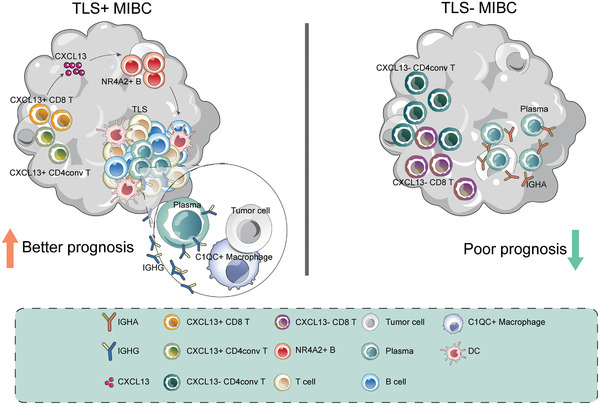
Proposed working model. In TLS‐pos MIBC, CXCL13+ T cells undergo expansion, leading to increased secretion of CXCL13. This cytokine interacts with the CXCR5 receptor on NR4A2+ B cells, facilitating TLS formation. The plasma cells generated in this microenvironment predominantly produce the IGHG antibody, which enhances the phagocytic activity of C1QC macrophages. In contrast, TLS‐neg MIBC is marked by a higher prevalence of CXCL13‐ T cells and IGHA‐subtype plasma cells. The presence of TLS induces stronger anti‐tumor immunity, leading to a better prognosis in MIBC.

This study highlights the essential role of B cells and TLS within the tumor microenvironment of MIBC. Our findings indicate that elevated levels of B cells and TLS are associated with improved prognoses in MIBC patients. Notably, CXCL13 plays a significant role in TLS formation in MIBC and has been demonstrated to reduce mortality risk across various MIBC cohorts. CXCL13 secreted by CXCL13+ T cells, facilitates the clustering of NR4A2 B cells, thus fostering TLS development. TLS generates more IGHG subtype plasma cells, enhancing macrophage phagocytic function and activating anti‐tumor immunity. Moreover, our research has resulted in the development of a novel TLS biomarker for MIBC that effectively predicts patient outcomes. Overall, our results substantially advance the understanding of TLS's role in MIBC (**Figure** **8**).

B cells and TLS are widely recognized as prognostic markers across the same cancers.^[^ ^4^ ^]^ However, large‐scale, cross‐cohort validation studies in MIBC remain limited.^[^ ^10^, ^11^, ^31^ ^]^ In this study, we used a large no ICB MIBC cohort to conduct survival analysis, confirming that elevated B cell levels are associated with improved survival outcomes in MIBC patients. Additionally, B cells have been significantly upregulated in ICB responders in melanoma,^[^ ^32^ ^]^ renal cell carcinoma,^[^ ^33^ ^]^ and sarcoma.^[^ ^34^ ^]^ Given that ICB only elicits durable responses in 20–25% of metastatic UC patients,^[^ ^1^ ^]^ selecting patients using appropriate biomarkers is crucial for optimizing ICB therapy. Our results demonstrate that B cells are the strongest predictors of response in the ICB‐treated UC cohort. While previous studies on the bladder cancer immune microenvironment have primarily focused on T cells, myeloid cells, and fibroblasts,^[^ ^35^ ^]^ our findings suggest that research efforts should increasingly prioritize B cells in bladder cancer immunology. Furthermore, as B cells are predominantly localized within TLS in urothelial carcinoma, TLS provides a practical clinical marker for quantifying B cell levels. Our hospital cohort further validated that TLS can serve as a prognostic marker in urothelial carcinoma patients, highlighting the translational potential and broad clinical utility of TLS.

Spatial transcriptomics is a powerful tool for investigating the composition and function of tumor‐associated TLS. However, to date, no studies have explored its application in MIBC.^[^ ^36^ ^]^ In this study, we employed ST to characterize the cellular composition of TLS in MIBC. Compared to renal cancer,^[^ ^37^ ^]^ the TLS in MIBC exhibited a higher proportion of CD8+ T cells, Tregs, NK cells, DC cells, and plasma cells, potentially indicative of immune activation. In the TLS regions, there is an observed increase in tumor‐reactive T cells and significant activation of immune signaling pathways. This phenomenon elucidates the positive correlation between TLS and the ICB response in MIBC.^[^ ^8^ ^]^ Cytokines play a pivotal role in the formation of TLS,^[^ ^38^ ^]^ with CXCL13 emerging as a critical factor specifically in MIBC. Furthermore, CXCL13 has shown strong prognostic value in cohorts with MIBC and UC. As a B cell chemokine, CXCL13 has also been demonstrated in other cancer types to effectively induce the formation of TLS.^[^ ^39^ ^]^ In a mouse ovarian cancer model, recombinant CXCL13 protein induced TLS formation and improved survival.^[^ ^40^ ^]^ Similarly, in a colorectal cancer liver metastasis model, intra‐tumoral injection of CCL19/CXCL13 promoted TLS formation.^[^ ^41^ ^]^ Future studies could focus on inducing TLS formation by enhancing CXCL13 expression as a potential therapeutic strategy for MIBC.

While TLS is known to reshape the immune microenvironment,^[^ ^5^ ^]^ its mechanism of action in MIBC remains unclear. In our study, T cells in TLS‐pos MIBC were more likely to differentiate into CXCL13+ T cells. Compared to CXCL13‐ T cells, CXCL13+ T cells expressed more tumor‐reactive markers and exhibited significant clonal expansion. Further analysis revealed that a higher proportion of CXCL13+ CD8+ T cells was associated with better prognosis, whereas CXCL13‐ CD8+ T cells correlated with poorer outcomes. CXCL13+ T cells have also been upregulated in other tumors responsive to immunotherapy^[^ ^21^ ^]^ and are positively correlated with tumor neoantigen reactivity.^[^ ^18^ ^]^ These cells secrete CXCL13, which targets CXCR5 on NR4A2+ B cells and may promote TLS formation. Our findings showed that plasma cells were enriched in TLS‐pos MIBC, producing more IGHG antibodies and fewer IGHA antibodies, a pattern linked to improved prognosis. Recent pan‐cancer analyses have indicated that plasma cells secrete more IGHG in tumors and more IGHA in normal tissues.^[^ ^42^ ^]^ Antibodies play a crucial role in the tumor microenvironment, particularly in mediating ADCP by macrophages.^[^ ^29^ ^]^ Our results demonstrated that C1QC+ macrophages were upregulated in TLS‐pos MIBC, exhibited enhanced phagocytic function, and polarized toward the M1 phenotype, amplifying the antitumor immune response. As a result, the presence of TLS is linked to stronger antitumor immunity and better prognosis.

TLS gene signatures have become a central focus in TLS research, with signatures already developed for colorectal cancer,^[^ ^43^ ^]^ breast cancer,^[^ ^44^ ^]^ gastric cancer,^[^ ^45^ ^]^ ovarian cancer,^[^ ^46^ ^]^ renal cancer,^[^ ^37^ ^]^ and other cancers.^[^ ^5^ ^]^ However, a defined TLS signature for MIBC has yet to be established. In this study, we identified a novel TLS‐specific gene signature, RMYY‐TLS, which includes CXCL13, CCL19, MS4A1, LTB, CD37, CORO1A, and IKZF1. RMYY‐TLS was successfully validated in two ICB UC cohorts and seven no ICB MIBC cohorts, with high RMYY‐TLS scores correlating with improved survival outcomes, particularly in the ICB cohorts. BCG is a standard immunotherapy for NMIBC,^[^ ^47^ ^]^ and the RMYY‐TLS signature also provides prognostic value for NMIBC patients undergoing BCG treatment. To further confirm the broad applicability of this signature, we validated its prognostic value in immunotherapy‐treated melanoma cohorts and renal cancer cohort. This study represents the largest multicenter effort to date in validating TLS as a biomarker in MIBC, underscoring the significant clinical translational potential of RMYY‐TLS.

This study underscores the pivotal role of B cells and TLS in MIBC. Higher levels of B cells and TLS are linked to a more effective antitumor immune response, resulting in significantly improved prognoses. Furthermore, the TLS‐specific gene signature identified in this study shows strong potential for clinical application. Overall, this research provides new insights into MIBC immunotherapy, highlighting the importance of TLS as both a biomarker and a therapeutic target.

## 4 Experimental Section

### Sample Collection

This study cohort comprised urothelial carcinoma specimens collected from Peking University People's Hospital between 2015 and 2024. The clinical follow‐up period for the patients spanned from the date of surgery until November 30, 2023. These samples were surgically resected, and pathologically confirmed. Fresh tissues intended for single‐cell sequencing were preserved in a tissue preservation solution, while bulk RNA sequencing samples were immediately stored at −80 °C. A portion of the samples was paraffin‐embedded and stored at room temperature. Informed consent was obtained from all patients before participation in the study. A total of 91 tumor samples were collected, and the detailed usage of each sample is provided in Table S2 (Supporting Information). This study involves human participants and was approved by the Ethics Committee of Peking University People's Hospital (ID: 2019PHB133‐01). Written informed consent was obtained from all patients and from all relatives of organ donors. All research procedures were conducted in accordance with the ethical standards of the Institutional Review Board.

### Collection of Non‐Immunotherapy Public Data

Seven bladder cancer cohorts were downloaded and curated from the Cancer Genome Atlas (TCGA, https://cancergenome.nih.gov/), the Gene Expression Omnibus (GEO, http://www.ncbi.nlm.nih.gov/geo/), and ArrayExpress (https://www.ebi.ac.uk/biostudies/arrayexpress). After screening, only MIBC samples with complete survival and RNA‐seq data were retained. The included cohorts were TCGA‐BLCA (*n* = 366), GSE13507 (*n* = 61),^[^ ^48^ ^]^ GSE31684 (*n* = 66),^[^ ^49^ ^]^ GSE32548 (*n* = 38),^[^ ^50^ ^]^ GSE32894 (*n* = 51),^[^ ^51^ ^]^ GSE48075 (*n* = 73),^[^ ^52^ ^]^ and E‐MTAB‐1803 (*n* = 73).^[^ ^53^ ^]^ Cohort‐6 refers to the combined non‐ICB MIBC datasets, comprising TCGA‐BLCA, GSE31684, GSE32548, GSE32894, GSE48075, and E‐MTAB‐1803. Furthermore, spatial transcriptomics datasets for renal cell carcinoma,^[^ ^37^ ^]^ hepatocellular carcinoma,^[^ ^54^ ^]^ and nasopharyngeal carcinoma were acquired.^[^ ^55^ ^]^

### Collection of Immunotherapy Public Data

IMvigor210 was a clinical cohort study investigating anti‐PD‐L1 therapy in patients with metastatic urothelial carcinoma (mUC).^[^ ^56^ ^]^ The data for UC patients were obtained from the R package “IMvigor210CoreBiologies,” and 298 samples were retained after screening. Additionally, the GSE176307 (*n* = 88) dataset, which includes an MIBC cohort treated with PD‐1/PD‐L1 inhibitors, was downloaded from GEO. GSE154261 represents a cohort of NMIBC patients treated with BCG, with data sourced from the GEO database.^[^ ^57^ ^]^ From the TIGER database (http://tiger.canceromics.org/), four melanoma immunotherapy cohorts were downloaded: GSE91061 (*n* = 101),^[^ ^58^ ^]^ phs000452 (*n* = 153),^[^ ^59^ ^]^ PRJEB23709 (*n* = 91),^[^ ^60^ ^]^ and GSE100797 (*n* = 25).^[^ ^61^ ^]^ The renal cell carcinoma cohort Braun_2020 (*n* = 311)^[^ ^62^ ^]^ was also downloaded. Cohort‐2 refers to the combined ICB‐treated UC cohorts from IMvigor210 and GSE176307.

### 4.1 scRNA‐Seq Workflow

To begin, single cells were isolated from fresh tissue samples. Using the 10x Genomics Chromium platform, single cells were encapsulated with barcoded gel beads within a microfluidic chip, creating Gel Bead‐in‐Emulsions (GEMs). Inside these GEMs, reverse transcription was performed to convert mRNA into cDNA, which was subsequently tagged with cell‐specific barcodes and unique molecular identifiers (UMIs). After the GEMs were lysed, the cDNA was released and subjected to PCR amplification to generate sequencing libraries. These libraries were then sequenced using the Illumina platform to obtain high‐throughput single‐cell transcriptome data.

### scRNA‐Seq Data Analysis

Single‐cell RNA sequencing data generated by the 10x Genomics platform were processed by aligning raw reads to the human genome (GRCh38) using Cell Ranger, generating a feature‐barcode matrix. Low‐quality cells (those with fewer than 500 genes, more than 5000 genes, or over 20% mitochondrial content) were filtered out to ensure data integrity. Data normalization was performed using the Seurat package, where the “NormalizeData” and “ScaleData” functions were applied to minimize technical noise. Harmony was employed to correct batch effects.^[^ ^63^ ^]^ Principal component analysis (PCA) was used for dimensionality reduction, followed by the “FindNeighbors” and “FindClusters” functions for unsupervised clustering. Visualization of the clustering results was achieved through UMAP or TSNE. Differential gene expression analysis was conducted using the “FindAllMarkers” function, and cell populations were annotated using a combination of classical marker genes and those from published literature.

### Single‐Cell TCR and BCR Prediction

The TRUST4 algorithm was employed to reconstruct immune receptor repertoires from single‐cell RNA‐seq data produced by the 10x Genomics Chromium platform.^[^ ^64^ ^]^ TRUST4 extracts candidate TCR and BCR sequences from FASTQ files, prioritizing assembly by abundance for increased speed and sensitivity. The resulting single‐cell transcriptomic data and VDJ recombination data were integrated into the R environment. Using the scRepertoire package,^[^ ^65^ ^]^ the “combineTCR” and “combineBCR” functions aggregated TCR and BCR sequences by cell barcode, allowing for the identification of clonotypes. These clonotypes were then integrated with scRNA‐seq data for further analysis and visualization using Seurat. Clonotypes with a frequency of two or more were considered clonal expansions, typically associated with tumor‐reactive cells.

### Pseudotime Analysis

To infer lineage and pseudotime from single‐cell RNA‐seq data, the Slingshot algorithm was applied.^[^ ^66^ ^]^ Initially, data reduced via UMAP were clustered using a Gaussian mixture model. Slingshot then constructed a minimum spanning tree based on the cluster centers to identify global lineage structures. Smooth branching curves were fitted along each lineage using a principal curve approach, allowing for pseudotime inference. Known biological start and end states were provided to guide the analysis and ensure biologically meaningful trajectory inference.

### Spatial Transcriptomics Sequencing

In order to profile the spatial gene expression within MIBC employed spatial transcriptomics technology. RNA was extracted from FFPE tissue sections, ensuring that the RNA Integrity Number exceeded 7. The sections were affixed to the capture area of a gene expression slide, followed by H&E staining and imaging. Tissue permeabilization was tailored to bladder cancer‐specific conditions, enabling the capture of mRNA from released cells via specific probes located in the microwells of the 10x Visium Spatial Gene Expression Kit. Library construction followed the 10x Visium protocol, with cDNA synthesis and amplification. The resulting cDNA libraries were subjected to high‐throughput sequencing on an Illumina platform.

### Spatial Transcriptomics Analysis

The raw Visium ST data were processed using 10x Genomics' Space Ranger software. The sequencing reads were aligned to the human reference genome (GRCh38), producing a matrix of feature barcodes. Data were subsequently scaled and normalized using the “scTransform” function to analyze highly variable features. To address batch effects across integrated ST samples, the Harmony R package was employed.^[^ ^63^ ^]^ Dimensionality reduction was performed through principal component analysis, followed by unsupervised clustering and data visualization using the “FindNeighbors,” “FindClusters,” and “RunUMAP” functions in the Seurat package. Additionally, convolution analysis was performed using SpaCET, leveraging in‐house scRNA‐seq data.^[^ ^67^ ^]^

### Bulk RNA Sequencing

The tumor tissue was lysed using TRIzol RNA Isolation Reagents, and total RNA was extracted. The NEBNext Ultra RNA Library Prep Kit for Illumina was employed to construct the sequencing library. Poly(A) mRNA was enriched using Oligo d(T) magnetic beads, followed by fragmentation of the mRNA. Subsequently, first‐strand and second‐strand cDNA synthesis was performed, and the resulting double‐stranded cDNA was purified. The cDNA library was then subjected to end repair, ligation of Illumina sequencing adapters, and PCR amplification to enrich the library DNA. Finally, PCR products were purified using magnetic beads, and the library quality was assessed with an Agilent Bioanalyzer to ensure a correct and consistent size distribution, with fragment sizes of ≈350 bp. The qualified library was sequenced using the Illumina PE 300 platform.

### Bulk RNA Data Analysis

Raw data quality was first assessed using FastQC to ensure high‐quality reads. Sequencing adapters were then trimmed with Cutadapt, and the cleaned reads were aligned to the GRCh38 reference genome using the STAR aligner. To standardize gene expression, TPM normalization was applied. Differential gene expression analysis was performed using DESeq2 to identify genes significantly upregulated or downregulated under varying conditions.

### Enrichment Analysis

Two enrichment analysis methods were employed: DE‐score and Gene Set Variation Analysis (GSVA).^[^ ^68^ ^]^ DE‐score quantifies gene expression enrichment by calculating the difference between the number of upregulated and downregulated genes within each enriched term. GSVA, a non‐parametric and unsupervised method, assesses pathway activity differences between samples based on gene set enrichment scores.

### Survival Analysis

The Kaplan–Meier method was used to evaluate the impact of various variables on survival time. The “survminer” package was utilized to determine the optimal cutoff for continuous variables, converting them into binary variables for more tailored analysis. Additionally, univariate and multivariate Cox regression analyses were conducted. Univariate Cox regression assessed the effect of individual variables on survival, while multivariate Cox regression evaluated the influence of multiple variables simultaneously, identifying independent predictors of survival.

### MCPcounter

MCPcounter was a transcriptome‐based computational method designed to robustly quantify the abundance of eight immune cell types and two stromal cell types.^[^ ^69^ ^]^ For each sample, abundance scores were derived from the gene expression matrix, facilitating direct comparison of the corresponding cell types across different samples. MCPcounter was also commonly used to score other gene sets.^[^ ^34^ ^]^ This approach allows for precise quantification of various cell populations and pathways within tissue samples, providing valuable insights into the tumor microenvironment and diseases such as cancer.

### HE Staining, Immunohistochemistry, and Immunofluorescence

In this study, hematoxylin and eosin (HE) staining, immunohistochemistry (IHC), and immunofluorescence (IF) were utilized to analyze tissue morphology, specific protein expression, and spatial distribution. All tissue samples were paraffin‐embedded. The preparation of paraffin sections involved fixation in 10% neutral formalin, dehydration through a graded ethanol series, clearing with xylene, and embedding in paraffin. Sections were then cut to a thickness of 4 µm. The HE staining procedure included deparaffinization, hydration, nuclear staining with hematoxylin, differentiation, counterstaining with eosin, graded dehydration, clearing, and mounting. Under the microscope, cell nuclei appeared blue, and the cytoplasm appeared red. IHC was employed to detect antigens in tissue sections using specific primary and secondary antibodies. The protocol included fixation, sectioning, antigen retrieval, blocking, incubation with the primary antibody, incubation with the secondary antibody, color development, and mounting. IF was used to detect multiple antigens simultaneously, following a procedure similar to IHC but utilizing fluorescently labeled secondary antibodies, with DAPI used for nuclear counterstaining at the final step.

### Statistical Analysis

All statistical analyses were conducted using R (version 4.2.1). Comparisons between the two groups in the study were made using Wilcoxon rank‐sum tests, while correlation analysis was performed using Pearson's correlation. Statistical significance was defined as a *p*‐value of less than 0.05.

## Conflict of Interest

The authors declare no conflict of interest.

## Supporting information



Supporting Information

Supporting Information

## Data Availability

The data that support the findings of this study are available from the corresponding author upon reasonable request.
