# Supplementary material for: Tertiary Lymphoid Structures are Linked to Enhanced Antitumor Immunity and Better Prognosis in Muscle‐Invasive Bladder Cancer
Source: Adv Sci (Weinh). 2024 Dec 30;12(7):2410998. doi: 10.1002/advs.202410998 (PMC11831474; doi:10.1002/advs.202410998)
Supplement: Supplementary file 1 — Supporting Information [file ADVS-12-2410998-s001.docx]

**Supplementary Figure**

**Figure S1**


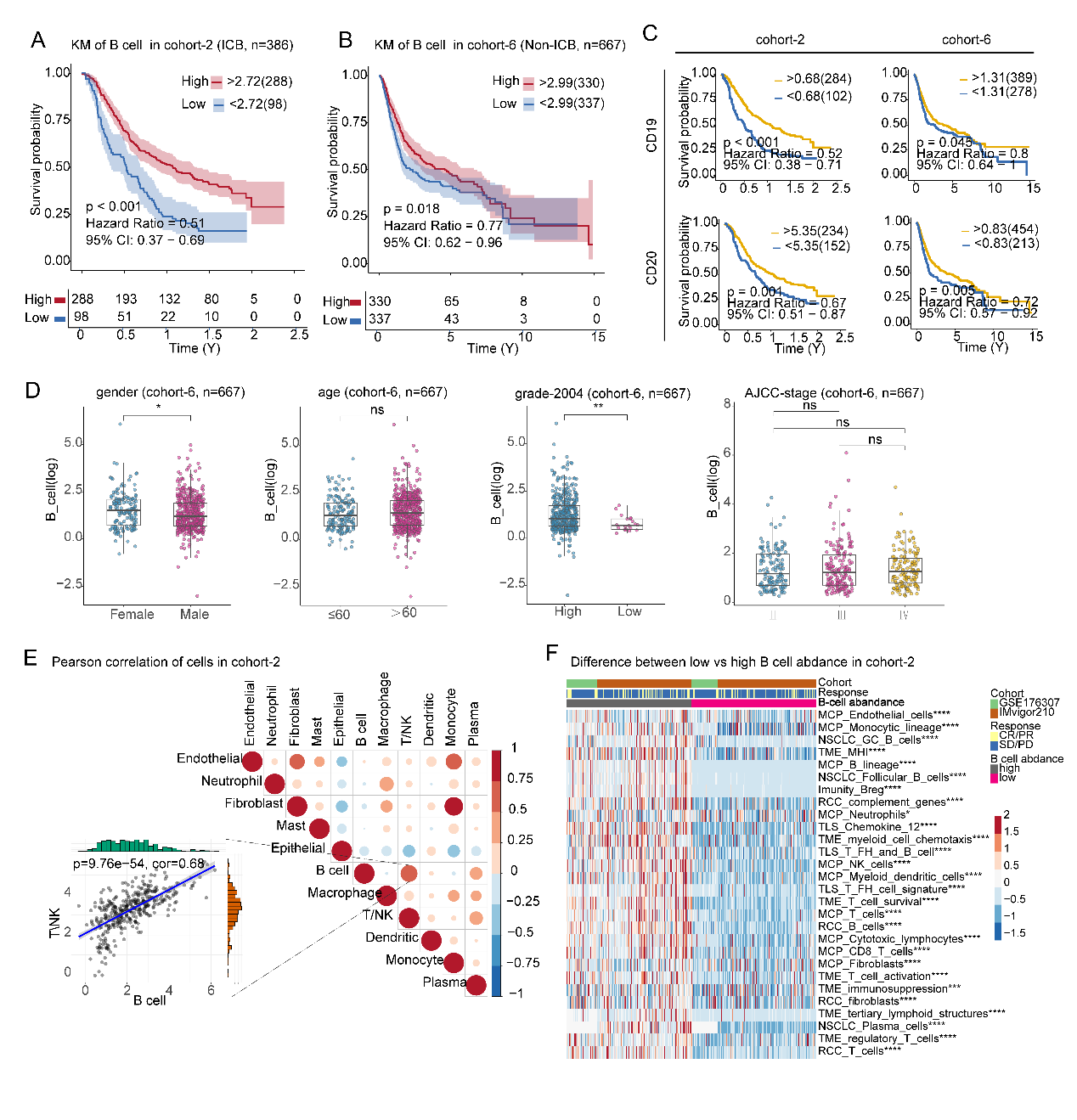


**Figure S1. Clinical Significance of B Cells**. A) Kaplan-Meier survival curves depicting B cell abundance in cohort-2. B) cohort-6. C) Survival analysis based on the expression levels of B cell markers CD19 and CD20. D) Analysis of differences in B cell abundance across various clinical conditions. E) Pearson correlation heatmap illustrating the abundance of various cell types. F) Heatmap displaying the differences in immune-related gene set scores between groups with high and low B cell abundance. *p < 0.05, **p < 0.01.

**Figure S2**


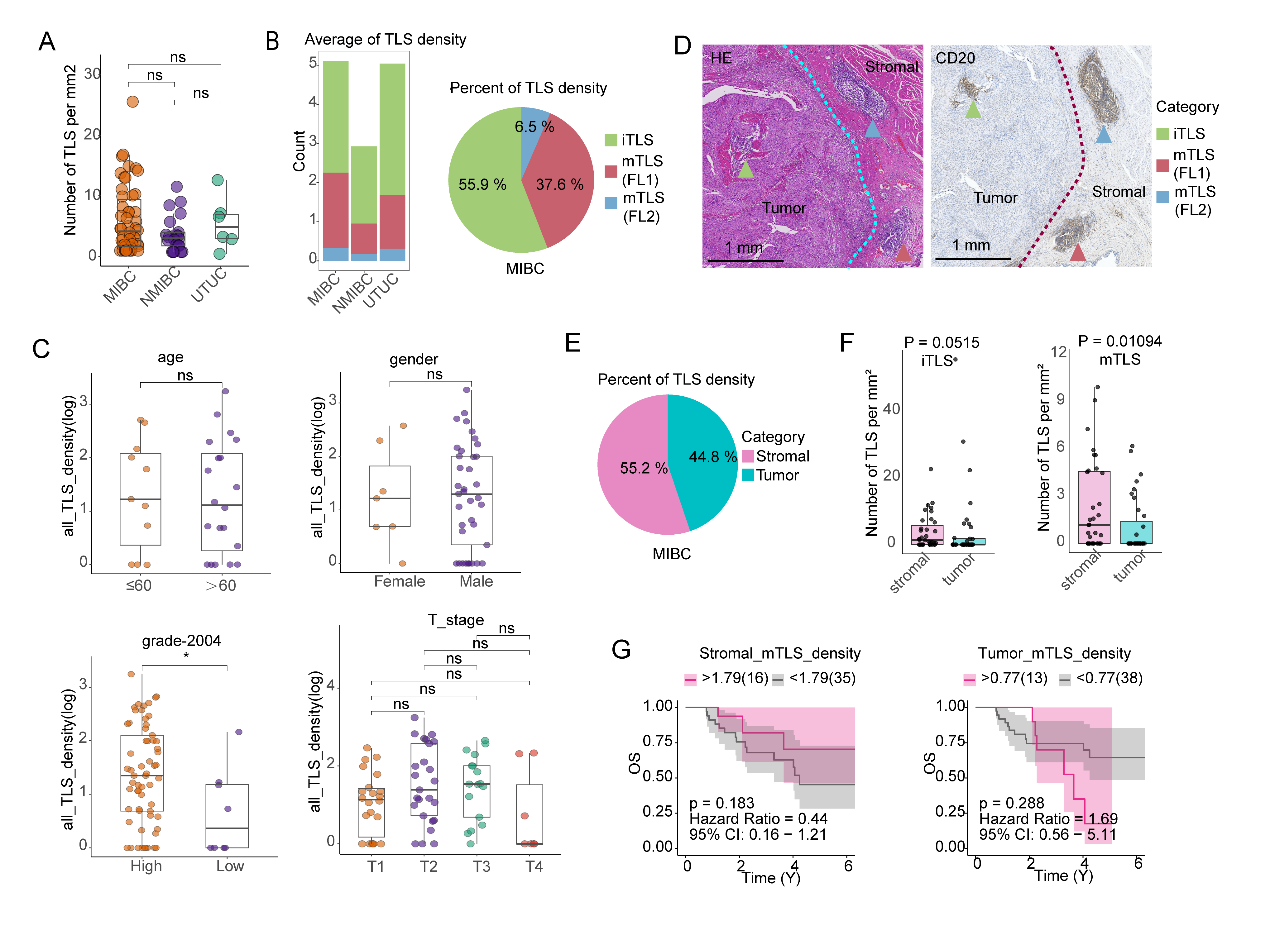


**Figure S2. Quantification and Distribution Heterogeneity of TLS.** A) Differences in TLS numbers across various UC subtypes. B) Average TLS density across UC subtypes, with a pie chart showing the proportion of TLS in MIBC. C) Comparative analysis of TLS density variations across different clinical conditions. D) Distribution of TLS in stromal and tumor regions. E) Proportion chart depicting TLS density in stromal versus tumor regions. F) Comparison of iTLS and mTLS distribution between stromal and tumor regions. G) Kaplan-Meier survival analysis based on mTLS density in stromal and tumor regions. *p < 0.05.

**Figure S3**


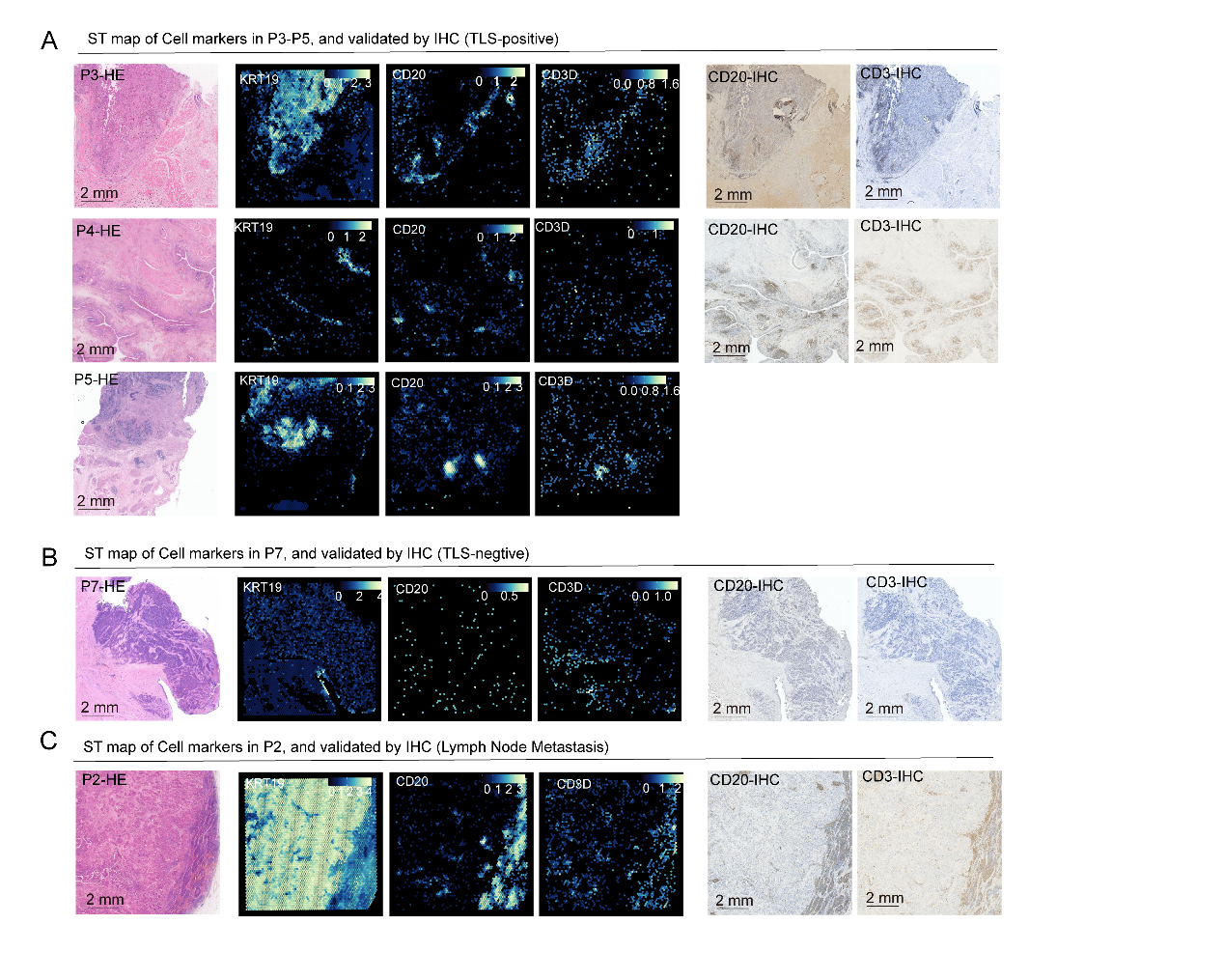


**Figure S3. Gene Expression and IHC Validation in ST Samples.** A) HE staining and spatial expression maps of marker genes in samples P3-P5, along with IHC validation of CD20 and CD3. B) P7 sample. C) P2 sample.

**Figure S4**


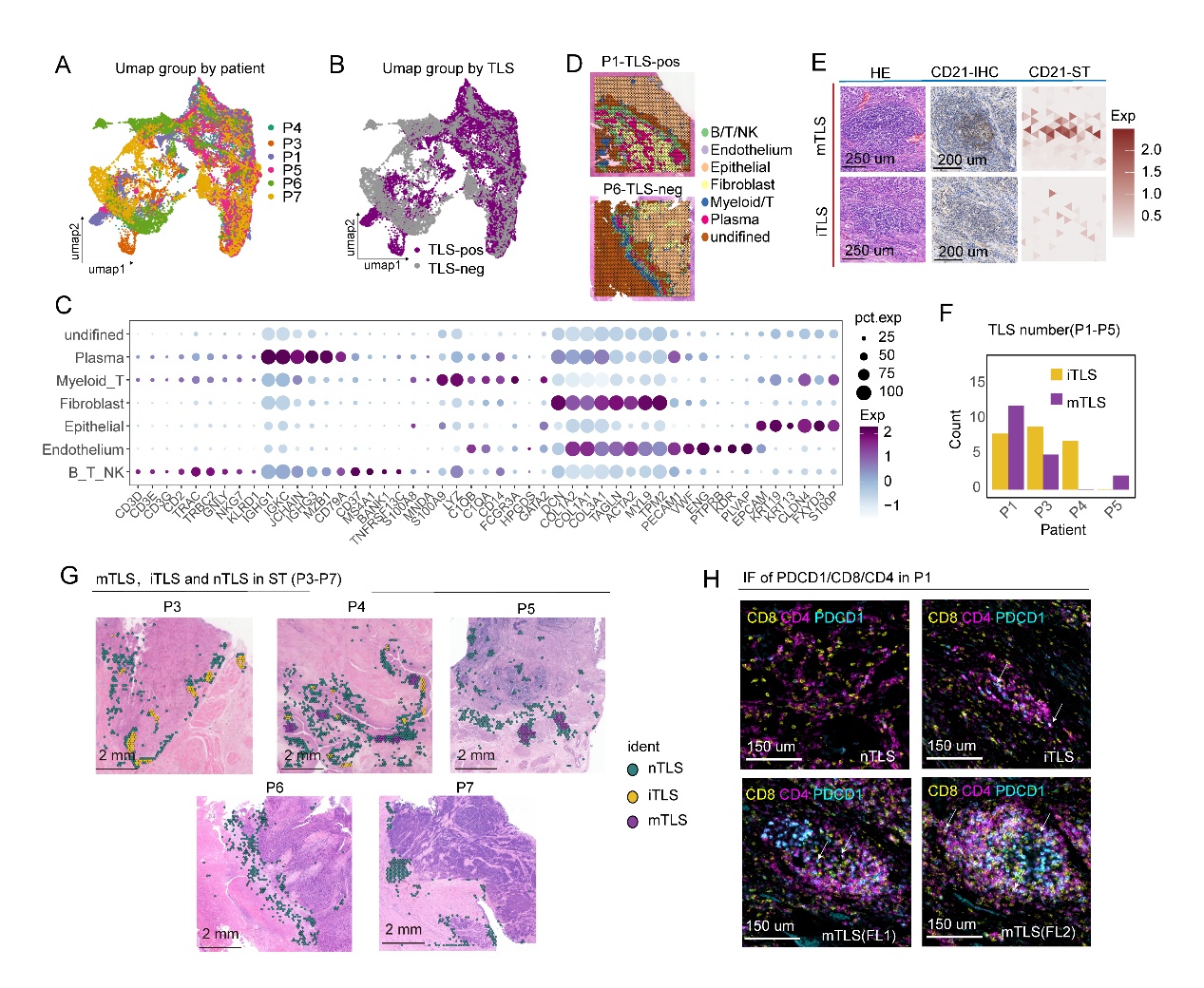


**Figure S4: Dimensionality Reduction and Clustering of ST Samples.** A) UMAP plots for different patients. B) UMAP distribution of TLS-pos and TLS-neg groups. C) Heatmap illustrating gene expression across different subpopulations. D) Subpopulation distribution in the P1 and P6 samples. E) HE staining, IHC, and expression analysis of CD21. F) Bar charts displaying the number of iTLS and mTLS in each sample. G) Distribution patterns of mTLS, iTLS, and nTLS in the P3-P7 samples. H) Multicolor immunofluorescence staining of CD8, CD4, PDCD1.

**Figure S5**


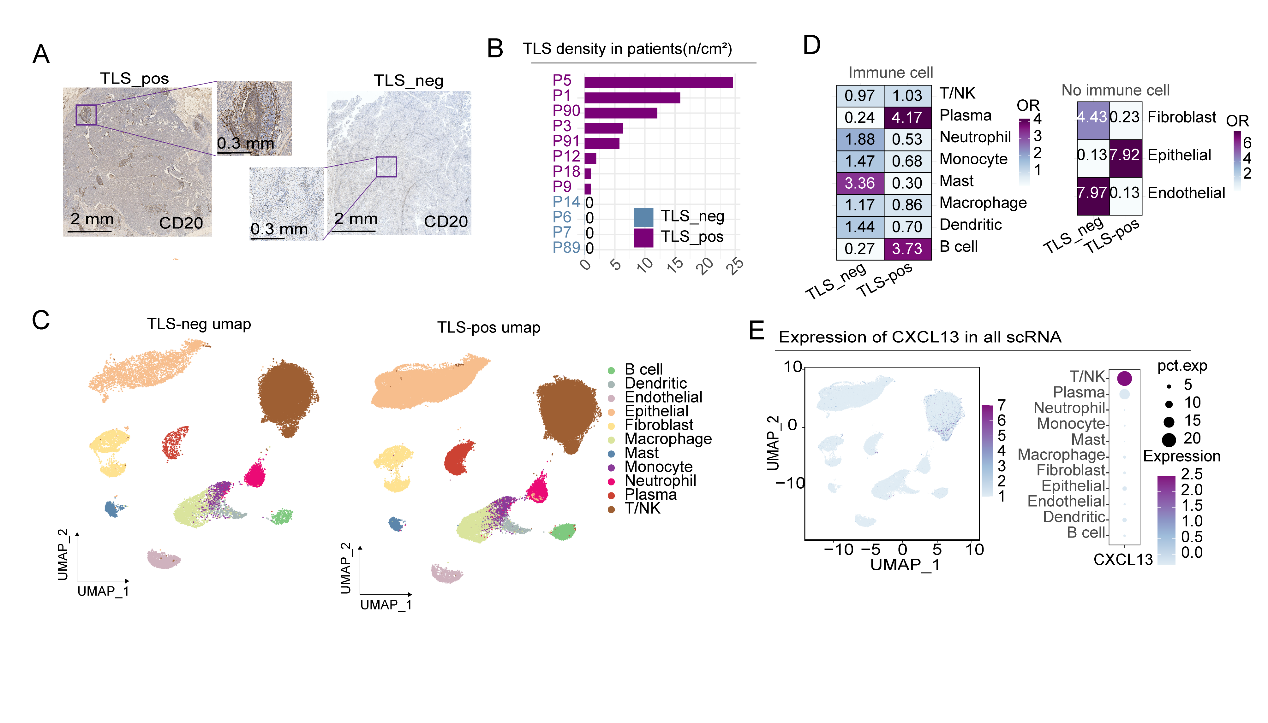


**Figure S5: Single-Cell Grouping Analysis of TLS-Pos and TLS-neg Samples.** A) IHC staining showing CD20 expression in TLS-pos and TLS-neg samples. B) Distribution of TLS density across different patient samples. C) UMAP plot illustrating cell subpopulations in TLS-pos and TLS-neg samples. D) Analysis of immune and non-immune cell distribution biases in TLS-pos and TLS-neg samples. E) On the left is the UMAP plot of CXCL13 expression, and on the right is the heatmap of CXCL13 expression.

**Figure S6**


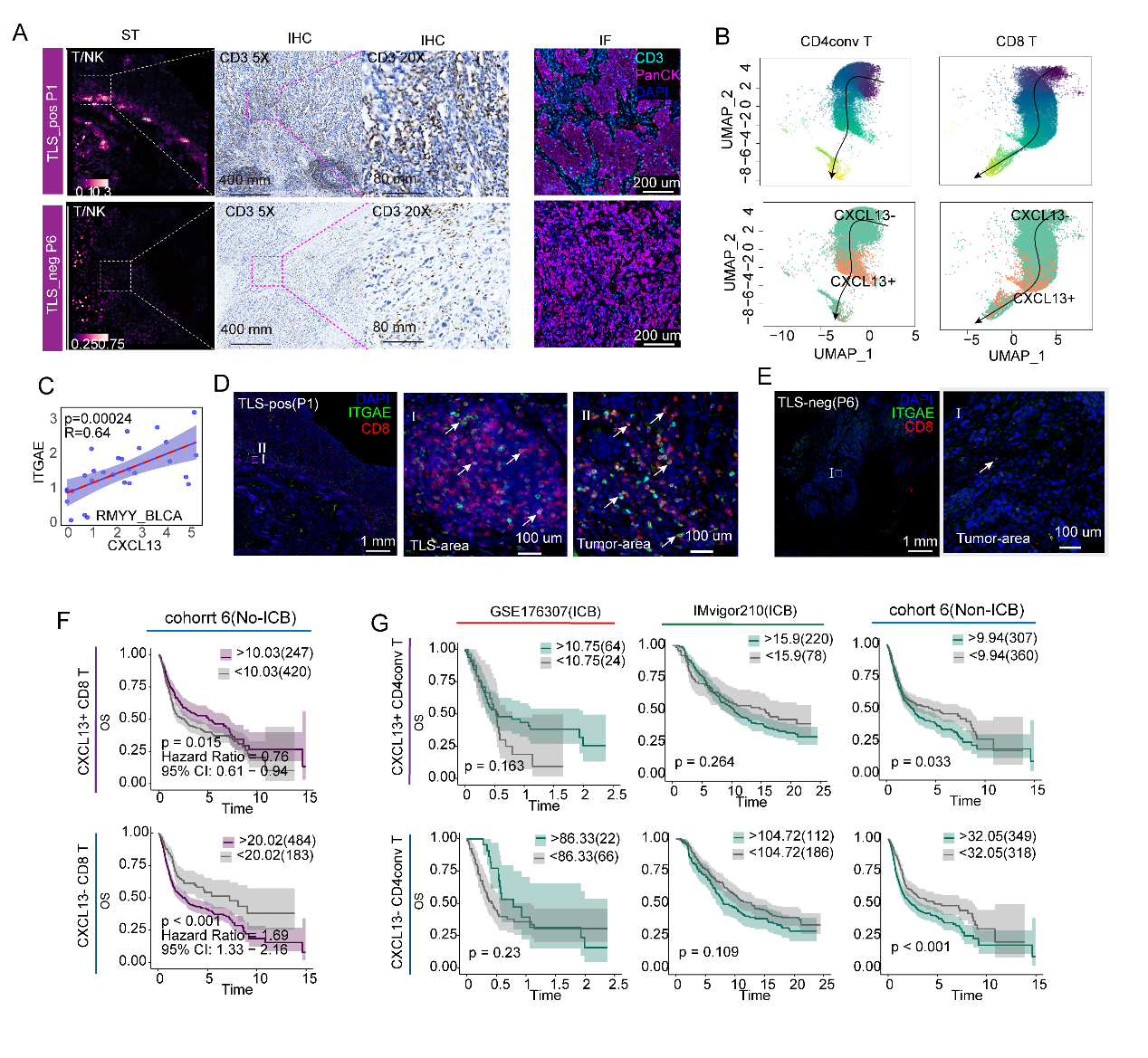


**Figure S6: Differences in T/NK Cells Between TLS-Pos and TLS-neg Samples.** A) ST convolution for T/NK cells, IHC and IF for CD3 T cell. B) UMAP plot showing cell developmental trajectories. C) Co-expression analysis of ITGAE and CXCL13 in the RMYY_BLCA cohort. D) Immunofluorescence staining illustrating ITGAE and CD8 expression in TLS-pos sample. E) TLS-neg sample. F) Survival analysis of CXCL13+ and CXCL13- CD8 T cells in the cohort-6 dataset. G) Survival analysis of CXCL13+ and CXCL13- CD4conv T cells.

**Figure S7**


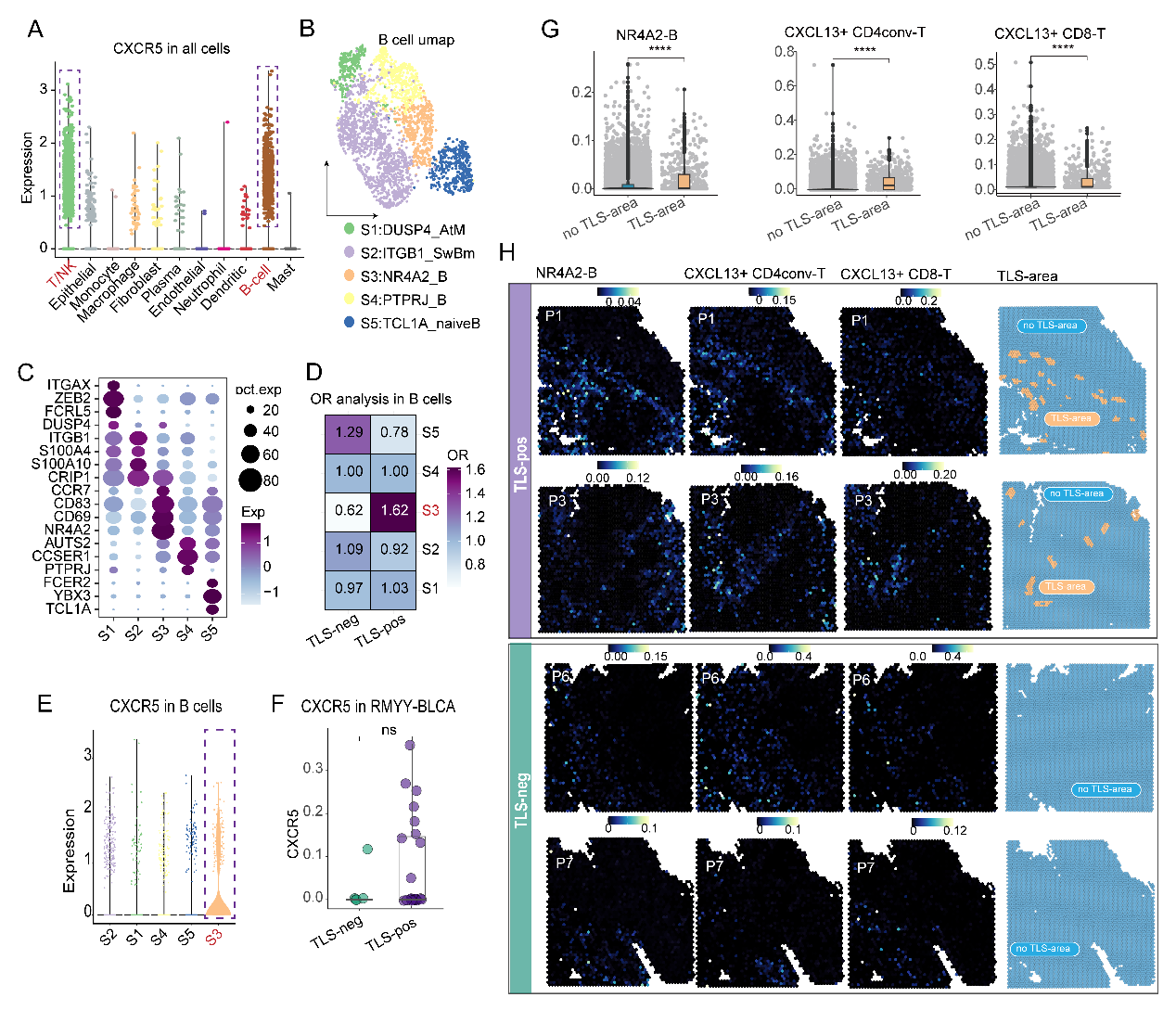


**Figure S7: Differences in B Cells Between TLS-pos and TLS-neg Samples.** A) CXCR5 expression in MIBC single-cell subpopulations. B) UMAP visualization of B cells. C) Heatmap of marker genes for different B cell subpopulations. D) Comparative analysis of B cell subpopulation distribution biases in TLS-pos and TLS-neg samples. E) CXCR5 expression in B cell subpopulations. F) CXCR5 expression in TLS-neg and TLS-pos samples. G) Comparison of cell content between TLS-area and no TLS-area using ST samples. H) The left displays cell deconvolution results, while the far right shows the spatial distribution in TLS areas. ****p < 0.0001.

**Figure S8**


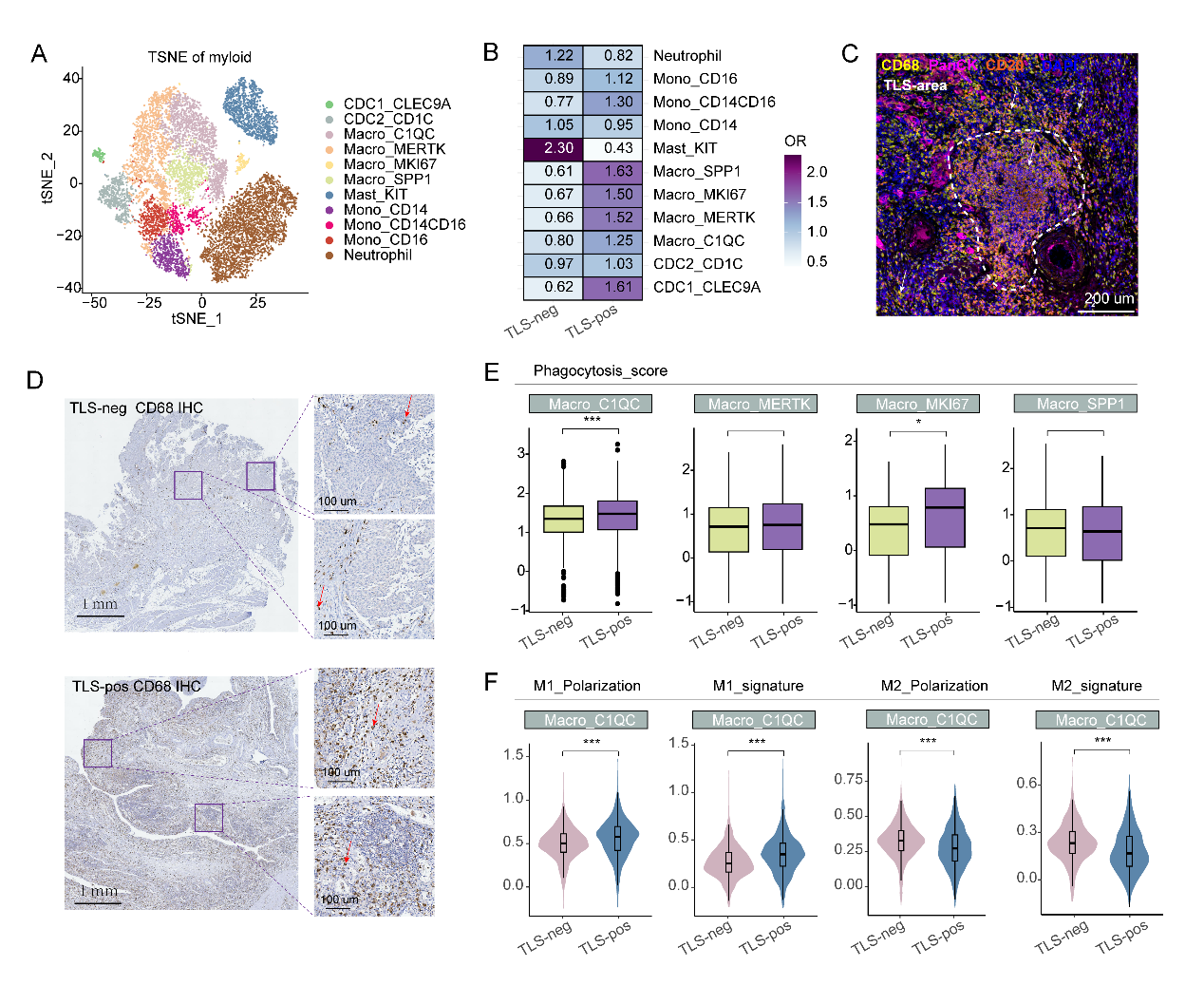


**Figure S8: Myeloid Cell Analysis.** A) TSNE plot illustrating myeloid cell subgroups. B) OR values comparing the distribution of various myeloid cell subgroups in TLS-pos versus TLS-neg samples. C) IF staining of the TLS region, including CD68, PanCK, and CD20. D) IHC staining of CD68. E) Box plot comparing phagocytic function scores among different macrophage subgroups. F) Violin plot depicting M1 and M2 polarization scores in Macro_C1QC macrophages. *p < 0.05, ***p < 0.001.

**Figure S9**


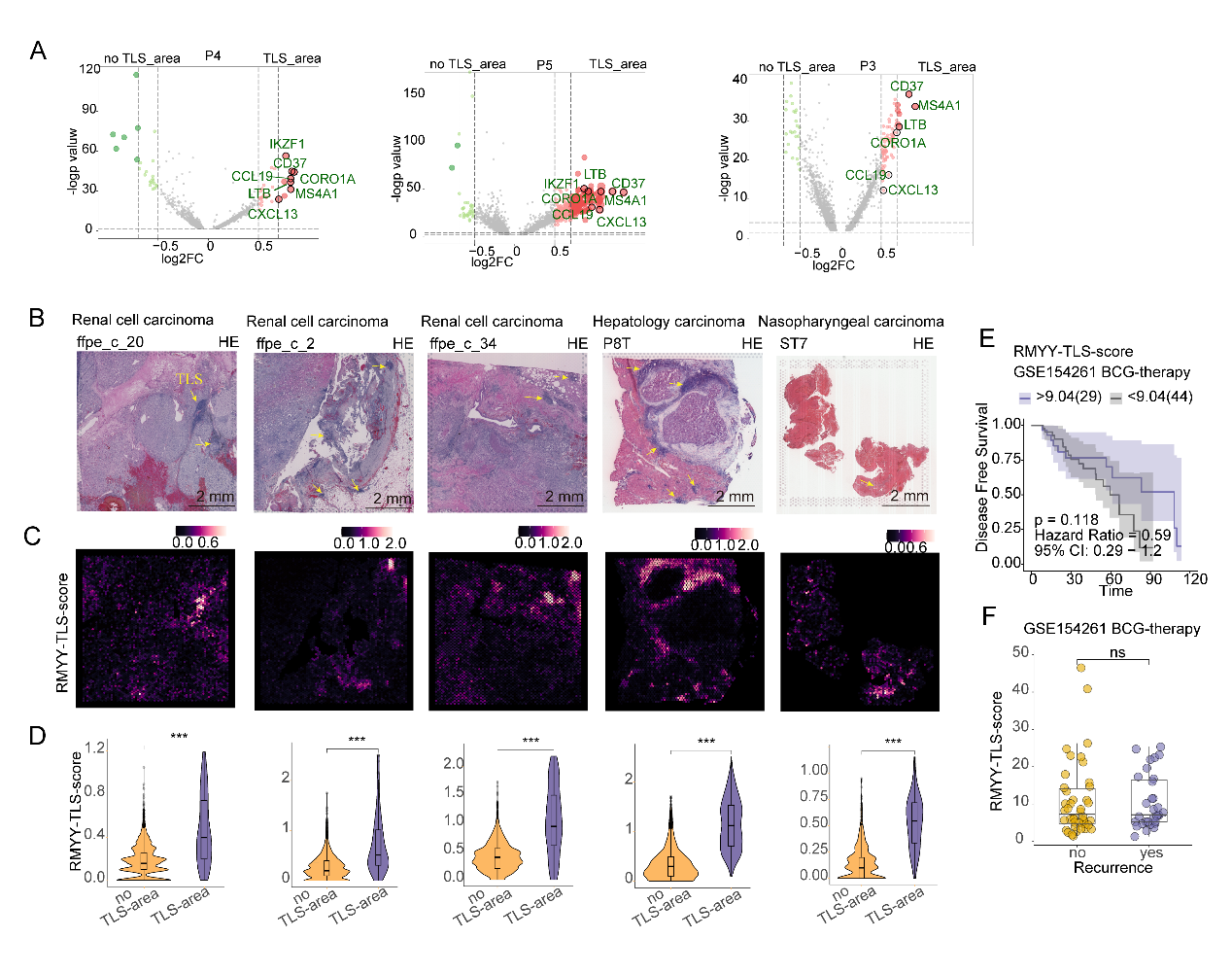


**Figure S9: Volcano Plots and RMYY-TLS Scoring.** A) Volcano plots showing differential gene expression between no-TLS and TLS areas in various samples. B) H&E staining images of control samples, with yellow arrows highlighting TLS regions. C) RMYY-TLS scores in ST samples. D) Comparison of RMYY-TLS scores between TLS-area and no TLS-areas. E) Kaplan-Meier analysis of RMYY-TLS in the BCG-treated cohort. F) Association between RMYY-TLS and tumor recurrence in the BCG-treated cohort. ***p < 0.001.
